# Nitric Oxide Synthase in the Central Nervous System and Peripheral Organs of *Stramonita haemastoma*: Protein Distribution and Gene Expression in Response to Thermal Stress

**DOI:** 10.3390/md13116636

**Published:** 2015-10-30

**Authors:** Mattia Toni, Federica De Angelis, Maria Carmela Bonaccorsi di Patti, Carla Cioni

**Affiliations:** 1 Department of Biology and Biotechnology “Charles Darwin”, Sapienza University, 00161 Rome, Italy; E-Mails: federica.deangelis@uniroma1.it (F.D.A.); carla.cioni@uniroma1.it (C.C.); 2 Department of Biochemical Sciences, Sapienza University of Rome, 00185 Rome, Italy; E-Mail: mariacarmela.bonaccorsi@uniroma1.it

**Keywords:** nitric oxide synthase, *S. haemastoma*, neogastropoda, thermal stress, gene expression, confocal microscopy, RT-PCR, western blot

## Abstract

Nitric oxide (NO) is generated via the oxidation of l-arginine by the enzyme NO synthase (NOS) both in vertebrates and invertebrates. Three NOS isoforms, nNOS, iNOS and eNOS, are known in vertebrates, whereas a single NOS isoform is usually expressed in invertebrates, sharing structural and functional characteristics with nNOS or iNOS depending on the species. The present paper is focused on the constitutive Ca^2+^/calmodulin-dependent nNOS recently sequenced by our group in the neogastropod *Stramonita haemastoma* (*Sh*NOS). In this paper we provide new data on cellular distribution of *Sh*NOS in the CNS (pedal ganglion) and peripheral organs (osphradium, tentacle, eye and foot) obtained by WB, IF, CM and NADPHd. Results demonstrated that NOS-like proteins are widely expressed in sensory receptor elements, neurons and epithelial cells. The detailed study of NOS distribution in peripheral and central neurons suggested that NOS is both intracellular and presynaptically located. Present findings confirm that NO may have a key role in the central neuronal circuits of gastropods and in sensory perception. The physiological relevance of NOS enzymes in the same organs was suggested by thermal stress experiments demonstrating that the constitutive expression of *Sh*NOS is modulated in a time- and organ-dependent manner in response to environmental stressors.

## 1. Introduction

Nitric oxide (NO) is a free natural gas that is used as signaling molecule by bacterial, animal and plant cells [1]. The biological effect of NO was discovered in mammals when this molecule was first identified as the endothelium-derived relaxing factor, a potent endogenous vasodilator [2,3]. A multitude of successive studies made it clear that NO is one of the most ancient signaling molecules involved in a myriad of physiological processes in metazoans, *i.e.*, blood pressure regulation, CNS signaling, defense, feeding response, environmental stress, hemocyte aggregation, learning, locomotion and swimming, memory, metamorphosis and symbiosis [4,5].

In metazoans, NO is enzymatically generated from the oxidation of l-arginine by NO synthases (NOS) that require NADPH as a cofactor. Three main NOS have been biochemically characterized and cloned in mammals: neuronal (nNOS or NOS1), endothelial (eNOS or NOS3) and inducible (iNOS) isoforms. Neuronal NOS and eNOS are Ca^2+^/CaM-dependent, constitutively expressed enzymes, whereas iNOS is a Ca^2+^-independent isoform expressed in activated macrophages and immune cells.

The three mammalian NOS isoforms are the products of distinct genes but share 50%–60% of their sequence identity and the binding sites for cofactors heme, BH4, CaM, FMN, FAD and NADPH. Nevertheless, each isoform has peculiar amino acid sequences that determine its distinctive catalytic and regulatory properties. Neuronal NOS is characterized by the presence of a PDZ domain at the N-terminal that allows its binding to other proteins and the targeting to specific sub-cellular domains [6,7]. The distinctive feature that differentiates iNOS from the other isoforms is the lack of the inhibitory loop located in the middle of the FMN-binding sub-domain that makes iNOS independent from calcium [8]. Endothelial NOS is specifically targeted to the caveolae of endothelial cells, and myristoylation at its N-terminal glycine and palmitoylation at cysteine residues 15 and 26 are required for its efficient localization to membranes [9].

NOS enzymes have been characterized and sequenced in a range of species belonging to all animal taxa (placozoa, cnidaria, arthropoda, mollusca, echinodermata and chordata) [5,10]. The three NOS known in mammals have been identified in all vertebrates with the exception of eNOS which is not present in fish [11]. By contrast, invertebrates have a single NOS isoform that has been identified as neuronal or inducible depending on the species. Most invertebrate NOSs are similar to the constitutive nNOS of mammals, except for their N-termini in which a PDZ domain is usually lacking [5].

According to a recently proposed evolutionary scenario, the three NOS isoforms would have arisen from a single ancestral gene by means of three rounds of genome duplication that occurred during early vertebrate evolution, whereas the diversity of invertebrate NOSs would be explained by occasional events of gene duplication [5]. Molecular characteristics of invertebrate NOSs are known in few species. Given the large number and diversity of invertebrates, new comparative data are required to investigate the presence of putative NOS orthologs and to corroborate the hypothesized evolutionary process.

The present paper focuses on mollusks. Among this large phylum, NOS proteins were fully sequenced in representative species belonging to three main classes: Gastropoda, Cephalopoda and Bivalvia. In particular, nNOS isoforms were fully sequenced in *Lymnaea stagnalis* (*Lym*-nNOS1, *Lym*-nNOS2, [12]), *Aplysia californica* (*Aply*-NOS and *Aply*-NOS2, [13,14]) and the terrestrial slug *Lehmannia valentiana* (*lim*-NOS1, [15,16]; *lim*-NOS2, [17]). A NOS full sequence was identified in the genome of the patellogastropod *Lottia gigantea* and in the cephalopod *Sepia officinalis* [5]. More recently, full nNOS was sequenced in the scallop *Chlamys farreri (Cf*NOS*)* [18] and its oxygenase domain was characterized in the oyster *Crassostrea virginica* [19]. Interestingly, *Cf*NOS has structural characteristics of a neuronal NOS but shares enzymatic features with both nNOS and iNOS.

Molecular characteristics of molluskan nNOSs are diverse in different species. Indeed, the N-terminal PDZ domain was found in nNOS proteins sequenced from *L. valentiana*, *L. gigantea*, *Stramonita haemastoma* and *C. farreri*, whereas the same domain is lacking in *A. californica*, *L. stagnalis*, *C. virginica* and *S. officinalis* [5]. The presence or absence of the PDZ domain may be functionally relevant, since this domain is involved in the intracellular localization and the corresponding physiological activity of the enzyme. To our knowledge, however, no studies were concerned with the role of the PDZ domain in molluskan NOSs.

Neuronal NOS was recently characterized by our group in the muricid *Stramonita haemastoma*, a diffuse eastern Atlantic/Mediterranean species belonging to Neogastropoda. [20]. Fully sequenced mRNA coded for a NOS isoform (*Sh*NOS) having 48% of homology with human nNOS and sharing similar regulatory domains with the mammalian nNOS. Similarly to vertebrate nNOS, *Sh*NOS is a constitutive Ca^2+^/calmodulin-dependent enzyme provided with the PDZ domain and the inhibitory loop and expressed in the nervous system.

In the present paper we provide further data obtained in the same animal model. Results are concerned with cellular distribution of *Sh*NOS in the central nervous system (CNS) and sensory organs. The purpose of the study was to contribute to the further understanding of functional roles of nNOS enzymes in mollusks. Given the unavailability of specific antibodies against molluskan NOS and considering the high degree of similarity between *Sh*NOS and mammalian nNOS, the immunohistochemical detection of nNOS in *S. haemastoma* was performed by using commercial antibodies against mammalian nNOS preliminarily tested by Western blot. Immunohistochemistry was performed on tissue samples from the nerve ring (pedal ganglion), osphradium and tentacle, including eye and foot, in which both *Sh*NOS gene expression and NADPHd activity were previously detected [20].

The effect of acute thermal stress on *Sh*NOS expression was also evaluated by RT-PCR to understand whether the *Sh*NOS gene can be regulated by environmental factors acting as stressors for aquatic animals. On the other hand, the involvement of NOS in the stress response is well known in mammals [21]. Results presented here confirm that NO may act as an effector molecule in physiological processes and environmental stress responses in mollusks.

## 2. Results and Discussion

### 2.1. Western Blot *(*WB*)*

**Figure 1:**
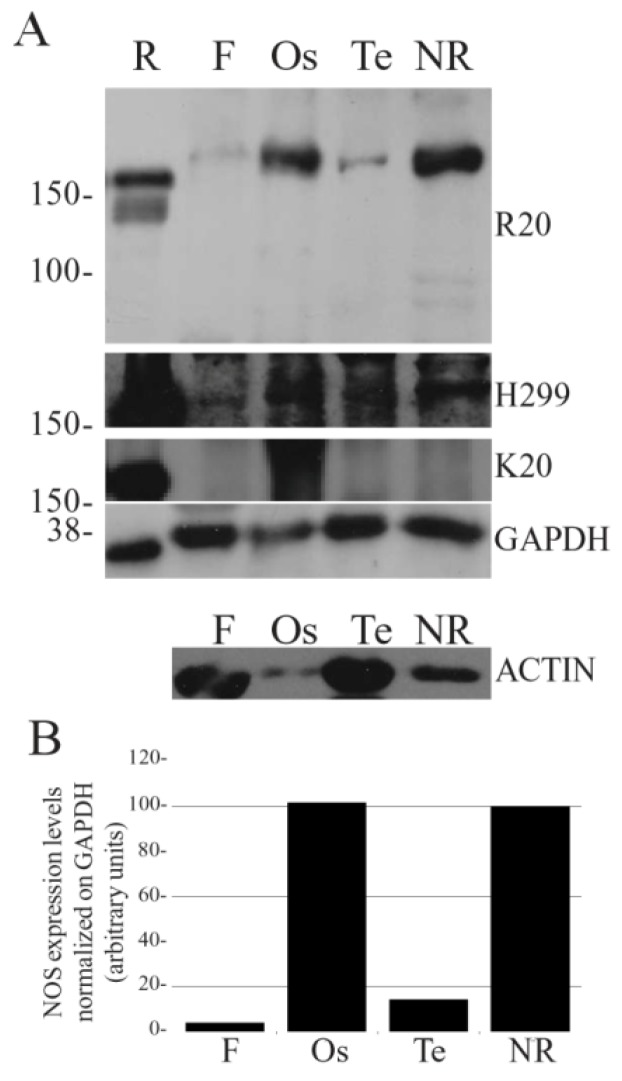
NOS expression in *S. haemastoma* organs. Western blot analysis of NOS expression levels in the nerve ring (NR), osphradium (Os), tentacle (Te) and foot (F) of *S. haemastoma* acclimated at 15 °C for 40 days. **A**: The NOS immunodetection was performed using three different NOS polyclonal antibodies (R20, H299 and K20) purchased from Santa Cruz Biotechnology, USA. Rat brain homogenate (R) was used as positive control. Anti-GAPDH and anti-actin antibodies were used on the same samples in order to normalize NOS expression. Standard molecular weights are indicated on the left. Each lane was loaded with 75 μg of protein homogenate. **B**: Band quantification of NOS expression levels detected by R20 antibody normalized on GAPDH levels.

Before analyzing NOS distribution, the crossreactivity of commercial anti-NOS antibodies against *Sh*NOS was tested by Western blot (WB). Three different polyclonal antibodies (R20, H299 and K20, Santa Cruz Biotechnology, USA) were tested on protein homogenates of *S. haemastoma* tissues and then used to assess levels of NOS-like protein expression in the foot, nerve ring, osphradium and tentacle (Figure 1). The use of antibodies directed against mammalian nNOS on molluskan tissues was justified by the moderate sequence homology between *Sh*NOS/rat nNOS (49.3%) and *Sh*NOS/human nNOS (48.3%). The specificity of the antibodies was evaluated on the basis of (a) molecular weight (MW), (b) intensity of immunolabeled bands and (c) level of background labeling. Among the three antibodies tested, both R20 and H299 intensely labeled a band at about 170 kDa that corresponds to the predicted *Sh*NOS molecular weight (169 kDa). However, R20 produced better results than H299 which revealed the presence of more intense background labeling (Figure 1A). Finally, K20 was not able to label defined bands in *S. haemastoma* tissues. For this reason, K20 was excluded and only R20 and H299 antibodies were used in IF experiments.

The densitometric analysis of R20 immunolabeled bands normalized on GAPDH revealed higher *Sh*NOS expression in the osphradium and nerve ring homogenates in comparison to tentacle and foot homogenates (Figure 1B). The level of *Sh*NOS expression in tentacle was about 10% of that detected in nerve ring and osphradium, whereas very low expression was found in foot samples.

These results are consistent with differences in *Sh*NOS gene expression detected by semi-quantitative RT-PCR in the same tissues [20] and confirm the presence of NOS enzymes in both the CNS and peripheral organs of *S. haemastoma*, suggesting a wide involvement of NO in cellular communication.

R20 and H299 antibodies tested in WB blot were used in IF and CM, in order to analyze *Sh*NOS cellular distribution in the same tissues. The observations demonstrated no significant differences in the distribution between R20 and H299 labeling.

Before proceeding with the IF analysis, histological observations were performed on serial sections of foot, osphradium and tentacle stained by hematoxylin-eosin to have basic information on their histological structure in *S. haemastoma*.

### 2.2. Osphradium: Histological Description

The osphradium is the molluskan sensory organ that perceives the water quality. It is situated in the roof of the mantle cavity, close to the siphon, and is mainly involved in chemoreception and mechanoreception. Although histological features of the osphradium in *S. haemastoma* are substantially similar to those of other neogastropods (*Buccinum undatum* [22], *Conus flavidus* [23], *Nassarius reticulatus* and *Nucella lapillus* [24]), they will be described here for the convenience of the reader.

**Figure 2:**
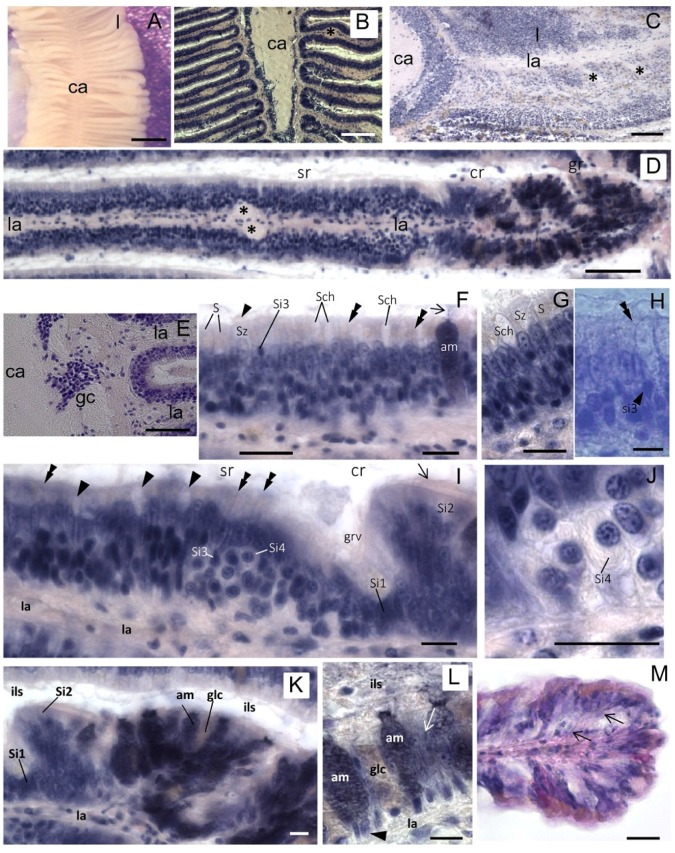
Osphradium histology. (**A)** Stereo microscope image of the osphradium that shows the bipinnate structure characterized by lamellae (l) radiating from the central axis (ca); (**B)** Frontal section of the osphradium showing the different cell density in the organ; (**C)** Sagittal section showing the lamellar axis (la) containing the lamellar nerve that sends a branch down (asterisks) into digitiform processes; (**D)** Frontal section of a single lamella showing its proximal-distal polarization characterized by sensory (sr), ciliated (cr) and glandular (gr) regions. Asterisks indicate the enlarged region in the lamellar axis (la) corresponding to the lamellar nerve branch observed in C; (**E)** Frontal section at the base of the lamella showing the lack of cell nuclei in the central axis and clusters of ganglionic cells (gc) at the base of the lamella; (**F)** Multilayered epithelium of the sensory region where S, Sz, Si3, Sch and am cells may be tentatively identified on the base of their morphology and position. The arrowhead indicates cilia on the apical portion of Sz cells. The arrow points to cell products secreted on the lamellar surface. Thin fibers running toward the epithelium surface can be glimpsed (double arrowheads); (**G)** Detail of the sensory epithelium in which yellowish granules in apical cytoplasm of S cells can be observed. Note the absence of these granules in Sz cells; (**H)** Apical process (double arrowhead) of a sensory Si3 cell (arrowhead) contacting the epithelium surface is shown; (**I)** Frontal section showing the lamellar groove (grv) that separates the sensory and ciliate regions. In the distal portion of the sensory region, Si4 cells can be observed. Sensory dendrites are regularly intercalated to epithelial cells on the apical surface of the epithelium (double arrowheads). Arrowhead points to Shc mucous cells. At the base of the groove, putative Si1 cells can be identified. Arrow points to the cilia of Si2 cells in the ciliated region; (**J)** Detail showing a Si4 cell with its shape at “halved onion”; (**K**) Image of neighboring ciliated and glandular regions showing the high concentration of gland cells in the latter; (**L)** Detail of the glandular region in which two different types of gland cells can be distinguished on the base of their hematoxylin staining: well-stained gland cells (am) and cells with low affinity for hematoxylin (glc). The nuclei are visible at the cell base (arrowhead). Gland cells secrete their products in the interlamellar space (ils). Among glands, support epithelial cells are present (arrow); (**M)** Frontal section of the lamellar tip where a different cell distribution can be observed. Thin fibers coursing from the lamellar axis to the epithelium surface are observed among epithelial cells (arrows). Bars: **A** = 500 μm; **B** = 150 μm; **C** = 100 μm; **D**, **E** = 50 μm; **F**, **G**, **I**–**L** = 10 μm; **H** = 5 μm; **M** = 25 μm.

As in other mollusks, the osphradium of *S. haemastoma* is a bipinnate organ consisting of two opposite rows of flat epithelial lamellae radiating from the central axis (Figure 2A–C). The osphradium contains the osphradial ganglion which has an inner neuropile surrounded by ganglionar cell bodies (Figure 2B). Ganglion cells are clustered in correspondence to the interlamellar spaces (Figure 2B,E). Osphradial lamellae radiate from the central axis and each lamella receives one branch of the lamellar nerve which forms its longitudinal axis (Figure 2C,D). Among lamellar nerve fibers, small neurons were aligned in the lamellar axis (Figure 2D).

Three main regions are distinguished in each osphradial lamella, the *sensory* (sr), *ciliated* (cr) and *glandular* (gr) region, similar to those described in *Buccinum* [22].

The *sensory region* (sr) (Figure 2D) corresponds to the proximal lamellar region. It is covered by a multilayered epithelium constituted by different cell types. According to Welsch and Storch’s histological description [25], the following cell types are distinguished by their position and morphology: S (Stutzzelle, supporting cell), Sz (Stutzzelle mit Zilien, supporting ciliated cell), Sch (Schleimzelle, mucous cell), Si1 (Sinneszelltyp1, type 1 sensory cell), Si2 (Sinneszelltyp 2, type 2 sensory cell), Si3 (Sinneszelltyp 3, type 3 sensory cell), Si4 (Sinneszelltyp 4, type 4 sensory cell).

In the sensory region of the *S. haemastoma* osphradium, supporting cells (S) with yellowish apical granules and supporting cells with cilia (Sz) were easily recognized (Figure 2F,G). Two types of mucous goblet cells (Sch) were interspersed between epithelial cells, having different affinity for hematoxylin (unstained and well-stained Sch) (Figure 2F,G,K,L). Well-stained Sch presumably correspond to the acid mucin cell (am) described by Hunt [26]. Both mucous cells discharge their secretion in the interlamellar spaces. Sensory cells were identified by a slender apical process terminating on the epithelial surface with a dilated ending (Figure 2H). Sensory endings were interspersed with epithelial cells on the apical surface of the epithelium (Figure 2I). Among sensory cells, putative Si3 and Si4 cells were recognizable. In particular, Si4 cells were easily identified by their “halved onion” shape in transverse sections, due to the presence of parallel cytoplasmic lamellae [24] (Figure 2I,J).

A small groove separates the sensory region from the *glandular region* (Figure 2I,K). The distal edge of the groove corresponds to the *ciliated region* which contains ciliated sensory Si2 cells, whereas sensory Si1 cells are located at the bottom of the groove (Figure 2I,K). The *glandular region* (gr) corresponds to the distalmost part of each lamella. It is lined by a single layer of supporting cells and contains abundant mucous cells (Figure 2K–M).

### 2.3. ShNOS Distribution in the Osphradium

**Figure 3:**
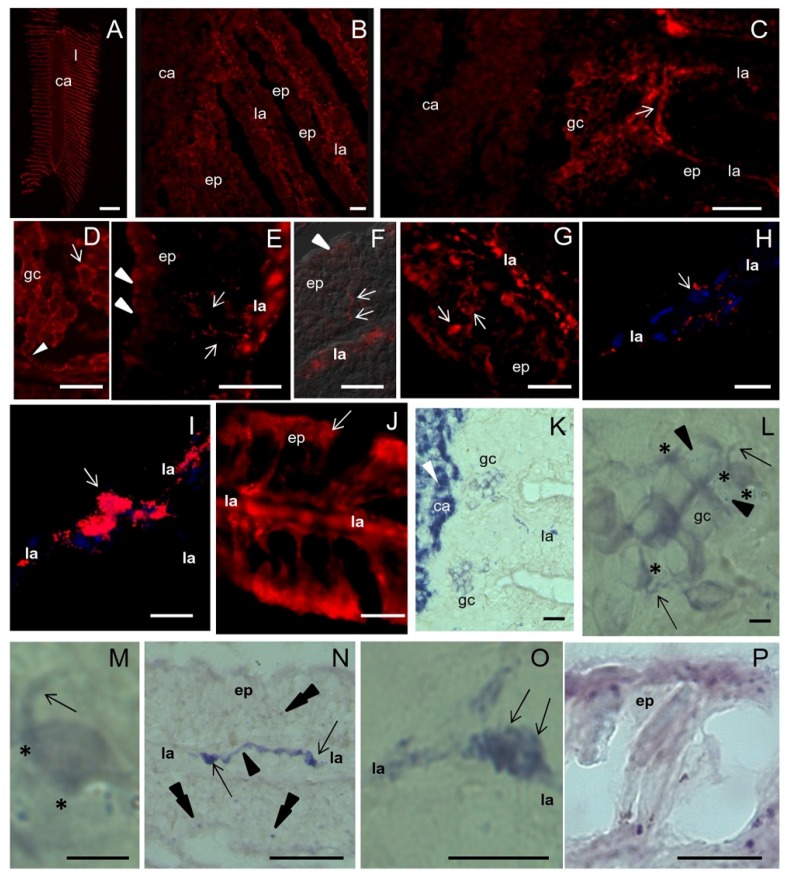
NOS distribution in the osphradium. **A**–**J**: IF and CM analysis with R20 antibody; **K**–**P**: NADPHd assay. (**A)** Frontal section of the osphradium showing intense NOS staining in the lamellae (l) and a weaker immunolabeling in the central axis (ca) of the organ; (**B**) Higher magnification showing the weak and diffuse NOS staining in the central axis corresponding to the osphradial ganglion, and the intense staining in the lamellar axis (la). A weaker labeling is also seen in lamellar epithelium (ep); (**C**) IF image showing NOS-IR ganglion cells (gc) and nerve fibers (arrow) at the base of the lamella. The different intensity of NOS labeling in the central axis and ganglionic cells can be appreciated; (**D**) Detail of ganglion cells showing an intense dotted staining along the edge of the cells (arrow) and a less intense labeling in the cytoplasm. Nerve NOS-IR fibers contacting ganglionic cells are seen (arrowhead); (**E**) Frontal section of the lamella. Strongly labeled cells are visible in the central axis. An intense IF signal is also present in the apical portion of the epithelial cells (arrowheads). Among epithelial cells, NOS-IR fibers coursing from the lamellar axis toward the epithelium surface are seen (arrows); **(F**) Superimposition of NOS staining to DIC image showing the path of NOS-IR fibers (arrows) among epithelial cells. Near the surface of the epithelium a dotted NOS staining is present (arrowhead); (**G**) NOS-labeled cells beneath the epithelium can be observed (arrows); (**H**,**I)** Two focal planes at different Z positions of the lamellar axis reveal the presence of NOS-positive spots (red signal) around NOS-unlabeled cells (compare the cell pointed by the arrow in H and I). Nucleic acids are labeled by TOTO3 (blue signal). Due to the large number of immunoreactive spots, these cells appear as NOS-expressing cells in epifluorescence; (**J**) IF microphotograph showing NOS-IR processes contacting the lamellar surface (arrow points to the dotted staining); (**K**) NADPHd showing the intense labeling in the central axis of the osphradium (arrowhead). Clusters of NADPHd-positive ganglionic cells are present at the periphery of the osphradial neuropile; (**L**) Microphotography of ganglionic cells showing the intense NADPHd staining at the periphery of the cells and few positive spots (arrowheads) in the cell bodies. NADPHd-positive nerve fibers (arrows) can be observed. NADPHd staining is more intense in the regions where nerve fibers contact the cell body (asterisks); (**M**) NADPHd-positive cells at high magnification showing their bipolar morphology; (**N**) Frontal section of the lamella showing NADPHd-positive neurons (arrows) and nerve fibers (arrowhead) in the central axis. Scarce positive spots are seen in the epithelium (double arrowheads); (**O**) Detail of a NADPHd-positive cell in the lamellar axis showing intensely labeled spots on its surface (arrows); (**P**) Sagittal section of the lamellar tip showing NADPHd-positive spots on the epithelium surface. Bars: **A** = 500 μm; **B**–**G**, **J**, **K**, **P** = 25 μm; **H**–**I**, **L**, **O** = 10 μm; **M** = 5 μm.

Intense NOS staining was found in the osphradial lamellae compared to the central axis of the sensory organ containing the voluminous ganglion (Figure 3A). Higher magnifications showed that the ganglion neuropile was little reactive for NOS (Figure 3B). In contrast, peripheral ganglion cell bodies were strongly labeled (Figure 3C). Immunoreactive products were distributed on these cells in small spots located at the cell periphery (punctate staining) whereas neuronal cytoplasm was weakly reactive (Figure 3D). This distribution suggested that *Sh*NOS is localized to axon terminals contacting ganglion neurons.

NOS staining was intense in the osphradial lamellae where small cells and thin fibers immunoreacted for NOS (Figure 3E–G). Immunoreactive cells were located in both the lamellar axis and subepithelial or intraepithelial position (Figure 3G). Positive fibers were scattered between unstained epithelial cells (Figure 3E–G) and were often continuous with discrete small NOS-IR regions of the epithelial surface (Figure 3F,J). According to the histological description reported before, positive subepithelial/intraepithelial cells may be identified as sensory receptor cells and the regions labeled for NOS in the apical epithelium may represent their apical ending contacting the free surface (compare Figure 3E to Figure 2H). Sensory receptor cells with similar shape and position were already described in *Thais* (=*Stramonita*) *haemastoma* [27] and this sustains our present identification.

Intense NOS staining was also found in small neurons located in the lamellar axis. Observations by CM at different Z positions clearly showed that their cytoplasm was NOS negative but their surface was completely covered by intensely immunoreactive spots (Figure 3H,I). In this regard, epifluorescence images were misleading. Thus, neurons in the lamellar axis seem to receive abundant synaptic contacts from NOS-IR nerve fibers. Although the immunostainings did not reveal the origin of NOS-IR fibers innervating the osphradium, present observations suggest that *Sh*NOS is expressed in both sensory receptor cells, nerve fibers and axon terminals. Thus, a role for NO in the sensory reception is likely. In addition, the putative synaptic localization of *Sh*NOS suggests a role for NO at presynaptic level.

NOS staining was generally intense in the apical region of the lamellar epithelium (Figure 3E,J) where secretory granules are accumulated in mucous and epithelial cells. This is particularly evident in the distalmost part of the osphradial lamella (compare Figure 3J to Figure 2M). This evidence suggests that NOS enzymes are also actively expressed in secretory cells (for a further discussion of this aspect see Section 2.7).

NOS staining was compared with the NADPHd staining described in our previous study [20] and new observations were made where needed. Results showed a good overlapping of the two staining types, except in the central neuropile, where NADPHd staining was much more intense than NOS immunolabeling (compare Figure 3K to Figure 3B). This discrepancy may be explained by assuming that NOS activity is intense in nerve fibers, thus producing the strong NADPHd staining of the central neuropile. Indeed, IF is able to detect NOS enzymes regardless of their activity whereas NADPHd only reveals enzymatically active NOS regardless of its amount. Anyway, it cannot be excluded that additional enzymes with NADPHd activities might contribute to the elevated activity found in the osphradial neuropile. 

As in IF observations, ganglion neurons were stained by NADPHd (Figure 3K) and the staining was much stronger at the cell periphery than in the neuronal cytoplasm (Figure 3L,M). NADPHd reaction was also found in neurons and nerve fibers along the lamellar axis (Figure 3N). Higher magnifications confirmed the punctate pattern of NADPHd staining (Figure 3O).

NOS staining and NADPHd were also overlapped in the apical epithelium (Figure 3P).

On the whole, the distribution of NOS/NADPHd in the *S. haemastoma* osphradium is compatible with the presence of enzymatically active *Sh*NOS in sensory cells and afferent fibers to osphradial neurons. When activated, *Sh*NOS would be able to locally produce NO. These results thus suggest that NO is not only involved in sensory reception but also in the modulation of sensory input to first- or second-order neurons located in the lamellar axis and in the osphradial ganglion.

### 2.4. Tentacle and Eye: Histological Description

**Figure 4:**
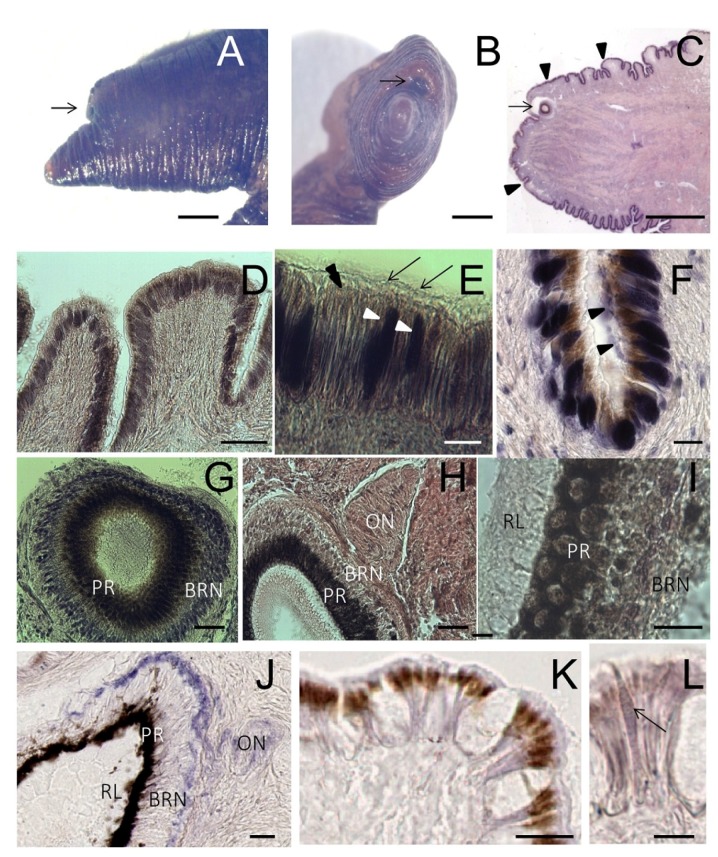
Tentacle: histology and NADPHd staining. (**A**,**B**) Lateral and frontal view of the tentacle observed at the stereo microscope. The arrow points to the eye; (**C**) Light microscopy of a sagittal section of the tentacle (stained by hematoxylin/eosin) showing the eye (arrow) and the folded surface (arrowheads); (**D**) Detail of the folded surface covered by a ciliated columnar epithelium; (**E**) Magnification of the epithelium showing the presence of cilia (arrows). Brownish granules are seen on the apical portion of the cells (double arrowhead). Dark gland cells are evident among epithelial elements (arrowheads); (**F**) Gland cells release their secretion on the tentacle surface (arrowheads); (**G**) Microphotograph of the eye where the basal retinal neurons (BRN) and the pigmented region (PR) are visible; (**H**) Sagittal section showing the optic nerve (ON); (**I**) Detail of RL contains distal segments of photoreceptors, PR contains retinal pigmented cells and BRN is formed by retinal neurons; (**J**–**L**) NADPHd assay on sagittal sections of the foot; (**J**) Microphotograph of the eye showing NADPHd blue staining in the optic nerve (ON), and the BRN layer. Intense NADPHd staining is present in the external BRN layer. The dark pigmentation of the PR prevents the detection of NADPHd staining; (**K**) Weak and diffuse NADPHd staining of epithelial cells. Apical pigments obscure NADPHd staining at the cell apex; (**L**) NADPHd-positive gland cells are seen within the epithelium (arrow). Bars: **A**–**C** = 500 μm; **D** = 50 μm; **E**, **G**, **H** = 10 μm; **I** = 5 μm; **F**, **J**–**K** = 25 μm.

The two eyes of *S. haemastoma* are bilaterally placed on the eyestalks, fused to the posterior edge of the cephalic tentacles. In stereo microscopy, tentacles appear dark brown because of the pigments contained in the epithelium (Figure 4A–C). Each tentacle is covered by a ciliated columnar epithelium provided with brown pigments and is rich in gland cells (Figure 4D–F). The presence of pigments, including carotenoids, indigoids, melanin, porphyrins and bilichromes, is a common feature in mollusk epithelia. 

The eye is a small hollow cup provided with a lens and surrounded by a capsule of connective tissue crossed by the optic nerve (on) (Figure 4G,H). The optic cup is lined by an internal *rhabdomeric layer* (RL) containing the distal segments of photoreceptors protruding into the eye cavity, a *pigmented region* (PR), formed by pigmented cells, and a *basal retinal neurons* (BRN) layer (Figure 4I).

### 2.5. ShNOS Distribution in the Tentacle and the Eye

**Figure 5:**
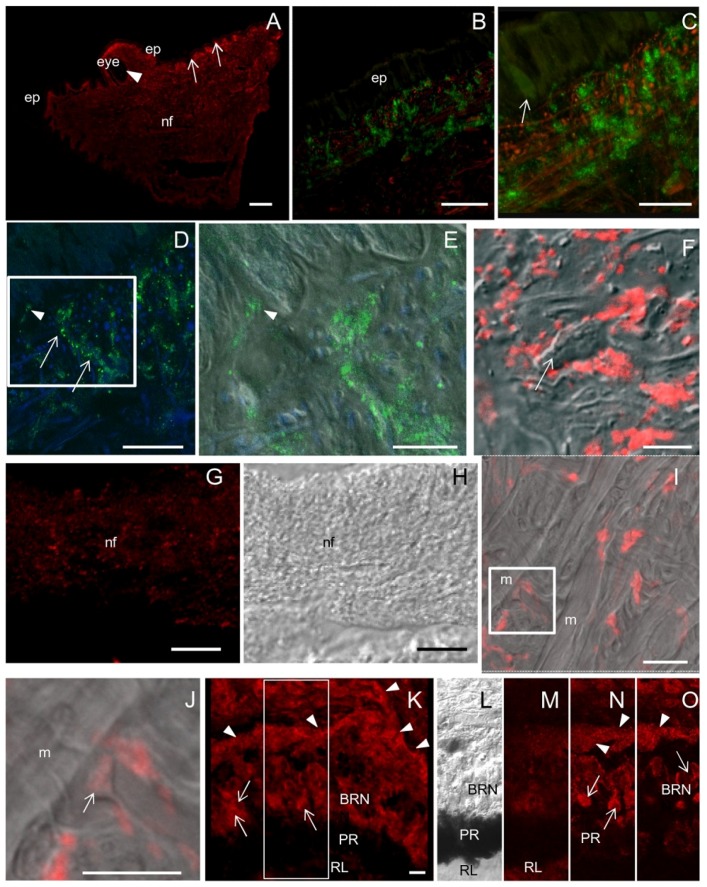
NOS distribution in the tentacle. **(A**) Sagittal section showing that NOS staining is more intense in the region beneath the epithelium (ep, arrows), in proximity of the eye (arrowhead) and in nerve fibers (nf); (**B**,**C**) CM double-staining with NOS antibodies (green) and phalloidin (red). Weakly NOS-labeled cells are seen in the epithelium whereas a dense network of NOS-IR nerve fibers are interspersed with phalloidin-positive actin filaments in the subepithelial layers. In the epithelium both gland (arrow in **C**) and epithelial cells are weakly labeled by NOS antibody; (**D**) CM double-staining with NOS antibodies (green) and the nucleic acid stain TOTO 3 (blue) to identify cell bodies. Dotted NOS staining are seen at the base of epithelial and gland cells (arrowhead) and along nerve fibers (arrows); (**E**) Superimposition of NOS/TOTO3 staining to DIC of the boxed area in **D**, showing NOS-IR fibers contacting the base of epithelial cells (arrowhead); (**F**) Superimposition of NOS staining (red) to DIC image clearly shows the presence of NOS-IR spots around unlabeled cells (arrow); (**G**) Detail of a nerve fascicle showing NOS-positive spots; (**H**) DIC image of panel G; (**I**) NOS/DIC superimposition showing the absence of NOS staining in muscle cells (m). Among muscle cells, NOS-positive spots are present; (**J**) Magnification of the boxed area in I. NOS-positive neuromuscular junctions can be observed (arrow); (**K**) NOS-IR cells (arrows) and nerve fibers (arrowheads) in the eye. (**L**) DIC image of the boxed area in J in which BRN, PR, and RL layers are recognizable; (**M**–**O**) Three focal planes taken at different z positions of the boxed area in **K** showing NOS-IR neurons (arrows) and nerve fibers (arrowhead) in the BRN. NOS staining is also detected in the RL and PR. **A**–**O**: R20 antibody. Bars: **A** = 200 μm; **B**, **C** = 50 μm; **D**, **G**, **H** = 25 μm; **E**, **F**, **I**, **K** = 10 μm; **J** = 5 μm.

A diffuse NOS staining was observed in the tentacle with a more intense labeling in the subepithelial region (Figure 5A). Triple CM staining by using NOS antibodies, phalloidin (for actin filaments) and TOTO3 (nucleic acid stain) showed that the subepithelial labeling was due to a dense network of varicose NOS-IR nerve fibers and phalloidin-positive fascicles of actin filaments (Figure 5B,C). Conversely, epithelial cells were weakly reactive for NOS (Figure 5B,C). NOS-IR varicosities were observed in close contact to the basal surface of epithelial cells (Figure 5D,E). The overlapping of NOS staining with DIC bright field clearly showed the presence of abundant NOS-positive spots around unstained cells (Figure 5F). The granular distribution of NOS immunostaining was also evident in large nerve fascicles (Figure 5G) and the varicose aspect of the nerve fibers was confirmed in the DIC pictures (Figure 5H).

These observations suggest that *Sh*NOS is associated with axonally transported membrane vesicles. This interpretation is supported by recent NADPHd/NOS ultrastructural studies in *Helix pomatia* and *Lymnaea stagnalis* that showed reactive products for NOS on both the membrane of agranular vesicles and the axolemma [28]. Ultrastructural observations are needed to elucidate this point in S. haemastoma. However, the presence of the PDZ domain in *Sh*NOS [20] might explain its association with vesicle membranes and the axolemma.

An examination by IF at CM demonstrated that muscle cells are not labeled by NOS antibodies (Figure 5I). This suggests that *Sh*NOS is not expressed in muscle cells and may represent a true difference between mollusks and mammals since human nNOS is localized beneath the sarcolemma in association with dystrophin [29]. However, the lack of NOS detection in muscle cells of *S. haemastoma* might be due to the existence of alternative spliced NOS isoforms that are not recognized by the NOS antibodies used in the present research. Additional nNOS isoforms, namely nNOSμ, originating from alternative splicing, were reported in murine skeletal muscle [30]. Otherwise, the inability of R20 and H299 antibodies to detect NOS in muscle cells might be due to interactions of *Sh*NOS with other proteins masking the epitope recognized by the NOS antibodies. Although muscle cells were NOS-negative, positive spots were observed at their periphery. The overlay of the NOS-IF signal on a single focal plane with the DIC image gave unambiguous identification of NOS-positive dilated terminals contacting unstained muscle cells. These terminals could be identified as neuromuscular junctions (Figure 5I,J) and their presence sustains the presynaptic localization of *Sh*NOS. Presynaptic nNOS at neuromuscular junctions may represent a common feature between mollusks and mammals. Indeed, nNOS was localized at the presynaptic level in mice neuromuscular junctions [31]. Literature data on mammals are quite conflicting on this point and other studies suggested that nNOS is rather accumulated at the postsynaptic level [32,33].

Intense NOS staining was also found in the eye. Cell bodies and axons of basal retinal neurons were strongly labeled, whereas the immunolabeling was much less intense in pigmented and rhabdomeric layers (Figure 5K–O).

The overall distribution of NOS labeling in the tentacle was consistent with NADPHd staining detected in the same organ [20,34]. New observations confirmed the intense NADPHd staining in the eye (Figure 4J) and the weaker staining in the tentacle epithelium (Figure 4K,L).

The distribution of *Sh*NOS indicates a role for NO in vision. To date, there is only sparse evidence of NOS enzymes in visual systems of mollusks. NADPHd staining was found in the optic nerve of *Aplysia* [35] and NOS enzymes were localized in the retina of *Loligo* [36] and *Sepia* [37] and in the eye neuropile and the optic nerve of *Bulla gouldiana* [38]. Immunohistochemical results in *S. haemastoma* are conflicting with those reported for *B. gouldiana* whose retinal neurons were not labeled for NOS. This discrepancy may be attributed to physiological differences between the two species or to the different sensitivity of antibodies (universal *vs.* nNOS antibodies) and detection methods employed.

The expression of NOS enzymes has been demonstrated in horizontal, amacrine and ganglion retinal cells of mammals [39,40] and non-mammalian vertebrates [41] and the involvement of NO in vision process may have been conserved during evolution.

### 2.6. Foot: *Histological Description*

**Figure 6:**
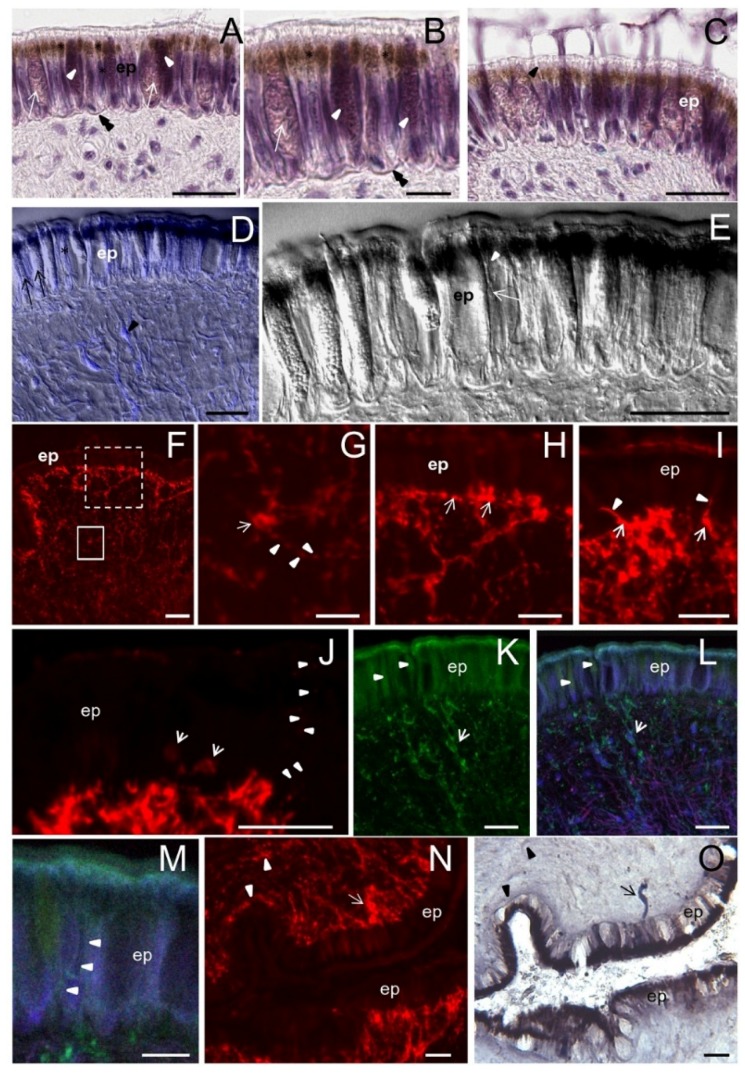
Foot: histology, NOS distribution and NADPHd staining. **A**–**E**: Histological analysis. **F**–**N**: NOS distribution analyzed by R20 antibody. **O**: NADPHd staining. (**A**,**B**) Sagittal section of the foot stained with hematoxylin/eosin. The ciliated columnar epithelium contains two types of gland cells with a different affinity for hematoxylin (arrows and arrowheads). Asterisks indicate pigment granules. The wavy basal lamina (double arrowhead) separates the epithelium from the underlying tissues; (**C**) Picture showing gland cells pouring their secretion on the foot surface. Slender cell processes are seen between epithelial elements (arrowhead); (**D)** The superimposition of TOTO3 staining to DIC allows a better appreciation of the cell morphology. Gland (arrows) and epithelial (asterisk) cells are visible. Beneath the epithelium, bipolar neurons are clearly seen (arrowhead); **(E**) DIC image at high magnification showing the presence of thin cell processes among epithelial elements (arrow). Cell processes contact the epithelial surface (arrowhead); (**F**) Sagittal section showing the distribution of NOS-like proteins beneath the epithelium; (**G**) Enlargement of the small boxed area in **F**. NOS-IR cells (arrow) and fibers (arrowheads) are seen; (**H**) Detail of the large dot-boxed area in **F**. NOS staining is intense at the base of the epithelium where NOS-IR cells and fibers are in close contact with epithelial cells (arrows). The epithelium exhibits a much weaker staining that is more intense in the apical portion; (**I**) IF image showing NOS-IR neurons (arrows) with processes penetrating the epithelium (arrowheads); (**J**) Detail showing intensely labeled NOS-IR cells in the subepithelial layers (arrows). Aligned NOS-positive spots toward the surface are visible (arrowheads) among epithelial cells, suggesting the presence of NOS-IR varicose fibers; (**K**,**L**) CM images of the same section confirming the wide distribution of NOS-like proteins beneath the epithelium. NOS-IR cells (arrows) and fibers are present. In the epithelium, a diffuse NOS labeling was observed in gland (arrowheads) and epithelial cells. The apical portion of epithelial cells shows an intense NOS-immunoreactivity. In **L** triple labeling with NOS antibodies (green), phalloidin (red) and TOTO3 (blue) is shown to better evaluate the distribution of NOS-positive staining; (**M**) Detail of the epithelium where the presence of NOS-IR thin processes (arrowheads) directed to the epithelium surface can be glimpsed among epithelial cells; (**N**,**O**) The same sagittal section of the foot stained with NOS-antibodies (**N**) and NADPHd assays (**O**). The two stainings overlapped in both cells (arrow) and cell processes (arrowheads). The number of NOS-positive elements was higher in IF than in NADPHd images. The detection of NADPHd reactivity in the epithelium was difficult due to the presence of natural pigments that obscure NADPHd staining. Bars: **A**, **C**–**E**, **G**–**O** = 25 μm; **B**, **M** = 10 μm; **F** = 50 μm.

The foot is the main locomotory organ of gastropods. It is coated by a ciliated columnar epithelium constituted by narrow and elongated cells (about 28 μm high) with brown granules at their apex (Figure 6A–C). A wavy basal lamina separates the epithelium from underlying tissues (Figure 6B). Two types of gland cells were interspersed among epithelial cells similar to those observed in other epithelia (Figure 6B,C). Among epithelial cells, slender processes of cells located in deeper layers are present (Figure 6C). Small bipolar neurons were identified by DIC in subepithelial layers innervating the base of the epithelium (Figure 6D). In DIC images, cell processes interspersed with epithelial cells are clearly seen (Figure 6D,E).

### 2.7. ShNOS Distribution in the Foot

A dense network of NOS-IR neuronal perikarya and axons were observed in subepithelial tissues of the foot (Figure 6F–I). NOS-IR axons were found terminating in close contact to the base of the epithelium (Figure 6H) or coursing between the epithelial cells (Figure 6I). A similar network of NADPHd-positive fibers was described in the radular sheath of *Helix pomatia* [42].

The foot epithelium was weakly reactive for NOS except at its apical surface, where pigment granules are accumulated (Figure 6I–J). A finest detection of NOS labeling by CM showed that both epithelial and mucous cells express NOS (Figure 6K–M) and confirmed the presence of thin labeled processes between the epithelial cells (Figure 6M).

As suggested for the epithelial lining of the osphradium, the intraepithelial NOS-IR processes may represent sensory processes of receptor cells located beneath the epithelium and their presence in the foot strengthens a putative role of NO in sensory functions. The conspicuous NOS innervation of the foot further sustains the involvement of NOS/NO in the perception of external stimuli.

Present findings also indicate that NO is somehow involved in pigment and mucous production. The presence of NOS enzymes appears to be a conserved feature of epithelial tissues, as part of the epithelial response to external agents. NOS expression was described in the mantle epithelium of *Lymnaea stagnalis* [43] and in the tentacle epithelium in *Aplysia californica* [44]. Other evidence correlated NOS with mucous glands, suggesting a role for NO in the release of granules. Indeed, NOS activity was detected by NADPHd in mucous cells of the foot in *Tapes philippinarum* [45] as well as in skin glands of domestic mammals [46]. 

The correspondence between NOS and NADPHd labeling was also analyzed in the foot by applying the two labelings to the same tissue section. As in other organs, we found a substantial overlapping of the two staining types, although only a minor part of the NOS-IR structures were also labeled by NADPHd (Figure 6N,O). NADPHd staining was detected in the epithelium, but the abundant brown pigments often prevented the localization of blue-colored reaction products (Figure 6O).

### 2.8. ShNOS Distribution in the Nerve Ring

The CNS of *S. haemastoma* consists of a circum-oesophageal ring of five paired ganglia: buccal, cerebral, pleural, pedal, and parietal ganglia. Our IF and CM analyses were focused on pedal ganglia whose NADPHd reactivity is intense, as described in our previous work [20].

**Figure 7:**
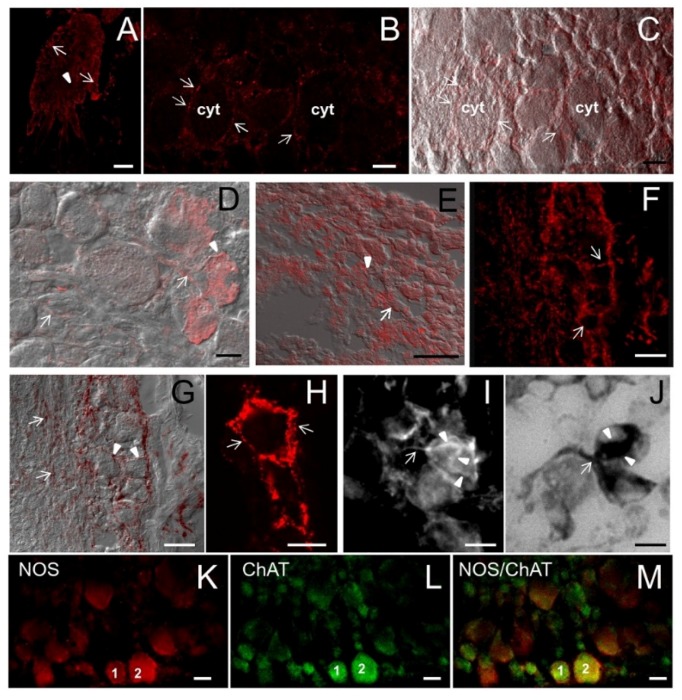
NOS distribution in the pedal ganglion. **(A**) Low magnification showing the gross distribution of NOS staining in the pedal ganglion. Both round neuron cell bodies (arrows) and nerve fibers (arrowhead) are labeled by NOS antibodies. (**B**) In this CM microphotography, the weak NOS staining in the cytoplasm (cyt) can be compared to the intense spotted staining on the neuronal surface (arrows); (**C**) Superimposition of NOS staining in **B** to the DIC image showing the position of NOS-positive spots in relation to cell morphology; (**D**,**E**) NOS/DIC image showing NOS-IR axons (arrows). NOS labeling appears particularly intense in the region where axons contact the neuron cell soma (arrowheads); (**F**) CM image showing the intense NOS staining around unstained neuron cell bodies (arrows); (**G**) Superimposition of NOS staining to DIC image allows us to better appreciate the intense NOS staining at the periphery of neuron cell bodies (arrowheads) and NOS-IR nerve fibers (arrows); (**H**) Detail of NOS staining in a single neuron: intense and defined NOS-labeled spots are present at the periphery of the cell (arrows); (**I**) IF microphotography showing NOS-positive neuronal processes (arrow) contacting one NOS-IR neuron. Note that NOS staining is diffuse and weak in the cell cytoplasm. NOS-stained neuronal processes have a basket-like distribution on the neuron cell soma (arrowheads). (**J**) NADPHd staining of the consecutive section to that in **I** containing the same neurons. A good overlapping of NOS and NADPHd staining can be observed; (**K**–**L**) Double-CM immunostainings of the pedal ganglion for NOS (**K**) and ChAT (**L**); (**M**) Overlay of fluorescence signals shown in **K** and **L** show that NOS and ChAT are co-localized in several neurons; the numbers “1” and “2” refer to the same neurons. **A**–**E**, **K**: M_R20 antibody; **F**–**H**: H299 antibody. **A**= 200 μm; **B**–**D**, **F**, **G**, **K**–**M** = 25 μm; **E**, **H**–**J** = 10 μm.

Both neuronal somata of pedal neurons and axosomatic terminals were stained for NOS (Figure 7A,B). CM of individual focal planes clearly showed weaker diffuse intracellular labeling compared to the intense punctate staining at the cell periphery, where immunoreactive spots were arranged in a “basket-like” fashion (Figure 7B,C). A similar distribution was described for the synaptic boutons on glandular cells of *Helix pomatia* [43], the salivary glands and cerebral ganglia of *Aplysia* [44], and the buccal ganglia of *Tritonia diomedea* [47].

To better appreciate NOS distribution, the immunostained sections were superimposed with DIC images and the resulting pictures confirmed that NOS labeling is particularly intense in nerve terminals (Figure 7C–E). H299 antibody was particularly sensitive in detecting NOS-positive spots at the cell periphery (Figure 7F–H).

The “basket-like” distribution likely corresponds to presynaptic NOS labeling and suggests that *Sh*NOS accumulates in afferent fibers innervating pedal neurons. We are inclined to this interpretation. However, the resolution of CM images did not allow us to determine whether NOS molecules are associated with pre- or postsynaptic terminals. Ultrastructural observations will contribute to clarifying this aspect.

NOS labeling was consistent with previous NADPHd staining in the same ganglion [20]. The degree of overlapping between the two staining types was evaluated in 10-μm-thick consecutive sections (containing the same neurons) double-labeled for NADPHd/NOS. Results clearly showed that both stainings are much stronger in nerve fibers contacting the cell soma than in the neuronal cytoplasm (Figure 7I,J).

To characterize NOS-expressing neurons in pedal ganglia, we have performed CM observations by double-immunostaining NOS/ChAT (choline acetyltransferase) (Figure 7K–M). A large number of pedal neurons express ChAT, demonstrating that ACh is involved in the motor control of the foot in *S. haemastoma*. Moreover, most pedal cholinergic neurons also express NOS. This finding indicates that NO exerts a modulatory role in cholinergic neurons.

### 2.9. Comparison of Western Blot with ShNOS Localization

NOS immunofluorescence showed that *Sh*NOS is highly expressed in all organs analyzed (osphradium, tentacle, foot and pedal ganglion). These data are not fully consistent with WB results, since quantitative differences in NOS expression were detected between different organs. This apparent contradiction can be explained by considering the tissue composition of the different organs. Indeed, WB was performed on total protein homogenates. Given the highest amount of NOS proteins expressed by nervous tissues, the percentage of NOS enzymes in each homogenate depends on its richness in nervous elements. According to this explanation, NOS enzymes are expected to be more concentrated in the nerve ring than in foot and tentacle homogenates where NOS-negative muscle tissues are abundant. Similarly, the high levels of NOS expression detected by WB in the osphradium may be explained by the presence of the large ganglion which is the main component of the organ.

In this sense, levels of actin expression in these tissues may be exemplifying. Actin is often used as housekeeping gene to normalize WB results. However, it could not be used in our experiments because it was much more expressed in foot and tentacle than in nerve ring homogenates due to their relative abundance in muscle cells (Figure 1).

### 2.10. Thermal Stress Effect on NOS Gene Expression in S. haemastoma

The wide distribution of *Sh*NOS suggested a key role for NO in nervous and epithelial tissues of *S. haemastoma*. With the aim of investigating the physiological relevance of the NOS/NO system in gastropods, we wondered whether the *Sh*NOS gene, which is constitutively expressed, can be regulated by environmental factors acting as stressors for aquatic animals. Our first approach was to analyze expression levels of the *Sh*NOS gene in response to thermal stress. Experiments were conducted by transferring *S. haemastoma* specimens from “home tanks” at 15 °C to “experimental tanks” at 28 °C for different time intervals comprised between 2 h and 24 h. mRNA levels were then analyzed by semi-quantitative RT-PCR in tissues expressing high levels of NOS proteins (osphradium, tentacle, foot and nerve ring).

On performing the experiments, special procedures were achieved for the animal transfer in order to minimize the stress of breaking them away from the substrate (see Materials and Methods). Anyhow, the effects due to the animal transfer were evaluated by performing two sets of controls: C1, animals acclimated at 15 °C for the duration of the experiment, and C2, animals transferred from home to experimental tanks at the same temperature, 15 °C. The comparison between C1 and C2 gave an evaluation of the transfer stress regardless of thermal response.

**Figure 8:**
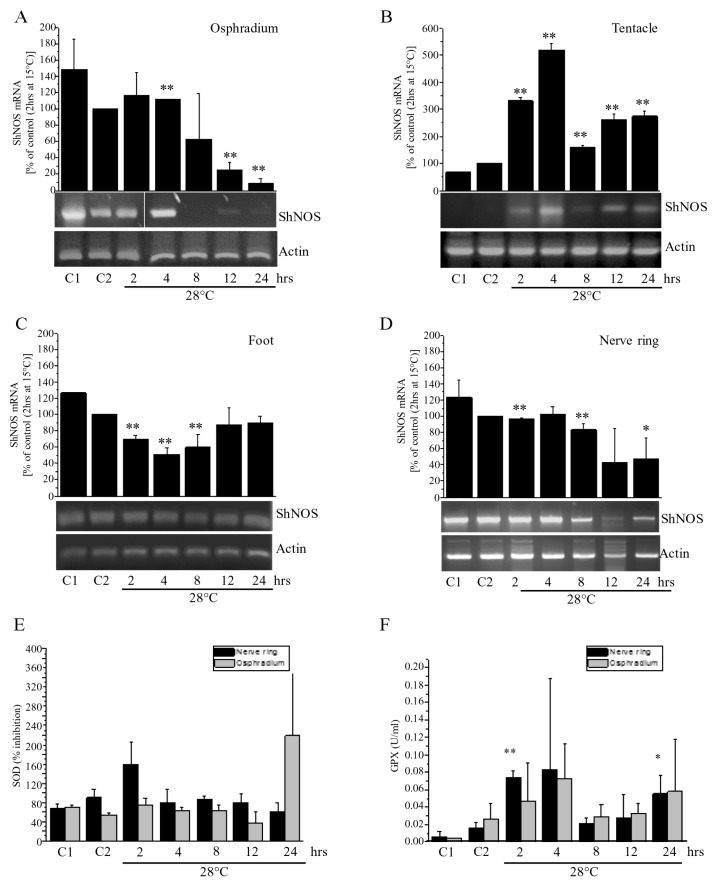
Effect of thermal stress on NOS gene expression and SOD and GPX activity in *S. haemastoma* organs. **A**–**D**: Semi-quantitative RT-PCR analysis of NOS gene expression normalized on actin performed in the osphradium (**A**), tentacle (**B**), foot (**C**) and nerve ring (**D**) samples of specimens placed at 28 °C for 2–24 h. C1 refers to control samples acclimated for 40 days at 15 °C and C2 refers to control samples transferred from the “home tank” at 15 °C to the “experimental tank” at the same temperature of 15 °C for 2 h. Band quantification was performed by Image J software; (**E**,**F**) SOD activity expressed as percentage of inhibition and GPX activity expressed as U/mL evaluated in the nerve ring (black bars) and osphradium (grey bars) subjected to thermal stress. The graph shows the average ± s.d. of three different experiments, performed under identical conditions. Asterisks indicate statistically significant difference (* = *p* < 0.05; ** = *p* < 0.02) between each sample and C2.

Levels of NOS gene expression were assessed by semi-quantitative RT-PCR by normalizing results on actin expression (Figure 8). Comparison between expression levels detected in C1 and C2 showed small variations in NOS gene expression in all samples that were statistically significant in only tentacle and foot (Figure 8B,C). Evidently, despite all precautions taken, mollusks are mildly affected by the transfer between tanks. In order to obtain information only related to temperature, NOS expression in thermally stressed samples was only compared with C2 controls.

In osphradium, foot, and nerve ring homogenates the thermal stress caused a reduction of *Sh*NOS gene expression, whereas an increase was observed in tentacle (Figure 8). In osphradium homogenates, a drastic and significant reduction in mRNA levels was detected 12 to 24 h from the beginning of the stress. In foot homogenates, NOS expression levels fell at 2–8 h, but returned to C2 control levels at 12 and 24 h. In the nerve ring, there was a decreasing trend in NOS gene expression, although values were not significant at each time (Figure 8D). A completely opposite trend was detected in tentacle homogenates (Figure 8B). Indeed, a significant increase in mRNA levels was detected from 2 to 24 h in comparison to lower levels of NOS expression found in C1 and C2. During the entire test duration, NOS levels showed variations, but they were always higher than those in C2.

Results demonstrated that *Sh*NOS gene expression is regulated in response to the rapid increase in temperature in a time- and organ-dependent way. Observed differences may depend on the different impact of thermal stress on specific functions. For example, in the tentacle (and eye), where *Sh*NOS is highly expressed, variations in light intensity or in the visual field during tank transfer might require an elevated demand of NO that is sustained by a strong increase in NOS gene expression.

Finally, despite all precautions taken, a comparison between C1 and C2 demonstrated that NOS gene expression varies in response to the transfer stress. This finding suggests that *Sh*NOS gene expression is regulated in response to different kinds of stressors.

### 2.11. Thermal Stress Effect on SOD and GPX Activity

Stress induced by water temperature changes has been associated with oxidative stress in aquatic organisms [48]. To investigate whether thermal stress can affect biological stress responses in *S. haemastoma*, the activity of SOD and GPX, two antioxidant enzymes involved in protection from oxidative stress, was measured. SOD removes superoxide by producing hydrogen peroxide and water, while GPX removes hydrogen peroxide by coupling its reduction to oxidation of glutathione. The levels of SOD and GPX activity were spectrophotometrically assessed in the osphradium and nerve ring of specimens subjected to thermal stress and compared with those detected in control conditions (Figure 8E,F).

Results obtained for SOD showed a peak of activity in the nerve ring at 2 h, followed by modest variations in both the osphradium and nerve ring compared to C2. The peak at 24 h measured in the osphradium is consistent with reports on the ark shell *Scapharca broughtonii* demonstrating an increase in SOD activity in the digestive glands and the gills of animals acclimated to 30 °C for 24 h [48]. GPX activity levels are low in C1 and C2, showing an increase at 2 and 4 h followed by a decrease to basal levels at 8 and 12 h and a second (lower) peak of activity was found at 24 h in both the osphradium and nerve ring.

Taken together, these results suggest that thermal stress can give rise to an antioxidant response in *S. haemastoma*. Changes in SOD activity are evident at 2 h in the nerve ring, while GPX activity is increased at 2 h in both the nerve ring and osphradium. GPX levels are still high at 4 h, suggesting that thermal stress can induce oxidative stress conditions that are effectively counteracted by increased SOD and GPX. Prolonged high temperature conditions leads to a rise in GPX activity at 24 h in both the nerve ring and osphradium. Thus, our results suggest that GPX may be a more sensitive marker of oxidative stress compared to SOD, in line with that reported in disk abalone (*Haliotis discus discus*) [49].

## 3. Experimental Section

### 3.1. Mollusk Collection and Maintenance

A total of 105 specimens of *S. haemastoma* were collected on the coast of Lazio (Italy) in September 2013. Upon arrival at the laboratory, mollusks were randomly allocated in 75 L (50 × 50 × 30 cm) indoor well-aerated glass aquaria (designated “home tank”) supplied with re-circulated seawater at 15 ± 1 °C with a final density of 35 specimens/tank. The inner surface of the tanks had been previously coated with removable plexiglass panels in order to make the inner glass surface of the tank inaccessible to mollusks and to oblige them to adhere to the plexiglass panels. This facilitated the transfer of animals from one tank to another during the experimental phases without removing mollusks from the substrate. Preliminary tests showed that *S. haemastoma* is able to adhere and move on plexiglass as on the glass wall. The mollusks were acclimated in home tanks at these conditions for 40 days.

### 3.2. Thermal Stress Test

In thermal stress experiments, mollusks were quickly moved from the “home tank” to the tank of similar size (designated “experimental tank”) containing seawater at the temperature of 28 ± 1 °C by transferring the plexiglass panels on which the animals were adhered. Mollusks were left in “experimental tanks” for a time ranging from 1 h 40 min to 23 h 40 min.

Two different control groups were used: mollusks acclimated at 15 °C in the “home tanks” (C1), and mollusks transferred to a tank filled with seawater at 15 ± 1 °C (designated “control tank”) for 1 h 40 min (C2). C2 allows us to evaluate the putative effect of the transfer regardless of the water temperature.

A total of seven different experimental groups consisting of 12 animals each were tested: C1, mollusk acclimated at 15 °C; C2, mollusk transferred in the “control tank” at 15 °C for 1 h 40 min; T1, at 28 °C for 1 h 40 min; T2, at 28 °C for 3 h 40 min, T3, at 28 °C for 7 h 40 min, T4, at 28 °C for 11 h 40 min, T5 at 28 °C for 23 h 40 min.

At the end of the thermal stress test, the mollusks were anesthetized for 20 min in a solution of MgCl_2_ (isotonic with seawater: 73.2 g MgCl_2_·6H_2_O per liter of distilled water) at 15 ± 1 °C (C1 and C2) or 28 °C (T1–T5), depending on the experimental group. Therefore, the total time of permanence at the test temperature was equal to the sum of the time spent in the “experimental tank” and in the anesthetic solution and amounted to 2 h (C2 and T1), 4 h (T2), 8 h (T3), 12 h (T4) and 24 h (T5).

Then, the shell was cracked and foot, nerve ring, osphradium and tentacles were dissected out at the stereo microscope. For each experimental group of 12 mollusks, tissues from seven animals were placed in RNAlater (Ambion, Austin, TX, USA) for the mRNA analysis, and tissues from the remaining five mollusks were frozen directly in dry ice and placed at −70 °C for protein and enzymatic analysis. 

The remaining specimens in the “control tanks” were fixed in Carnoys solution for 48 h and stored in 70% ethanol for histological investigation (five specimens), fixed in PFA solution (4% paraformaldehyde in 0.1 M phosphate buffered) for 24 h for immofluorescence analysis (five specimens) or frozen in RNAlater (Ambion, Austin, TX, USA) for Western blot.

### 3.3. Histological and Histochemical Processing and Evaluation

Specimens were dehydrated in increasing concentrations of ethanol (70%–99%) and embedded in paraffin (McCormick Scientific, Richmond, IL, USA) at 60 °C. Step sections with a thickness of 10 μm were cut, floated on deionized water at 45 °C, and mounted as single sections on Superfrost Plus glass slides (Menzel-Gläser, Braunschweig, Germany). Slides were subsequently dried at 60 °C for 24 h. Sections were stained in eosin and hematoxylin (Merck, Darmstadt, Germany). 

NADPH diaphorase histochemistry was performed as previously reported in [20].

Images were captured using a Moticam 2000 (2.0 megapixel) digital camera (Motic Instruments, Canada) and Motic Image Plus 2.0 analysis software. The final figure composition was done using Adobe Pagemaker 7.0 or Microsoft Office Power Point 2007 software.

### 3.4. Western Blot

Samples of foot, osphradium, tentacles and nervous ring from three specimens of *S. haemastoma* were homogenized in a denaturing lysis buffer containing urea (8 M urea, 50 mM Tris-HCl, pH 7.6, 0.1 M 2-mercaptoethanol, 1 mM dithiothreithol (DTT), 1 mM phenylmethylsulphonyl fluoride) and protease inhibitors (Roche, Mannheim, Germany) and the particulate matter was removed by centrifugation at 14,000 *g* for 20 min. The protein concentration was determined by the Lowry method [50]. For gel electrophoresis, proteins were denatured after boiling in Laemmli Sample Buffer for 5 min. Then 75 μg of proteins was loaded in each lane and separated in 8% SDS-polyacrylamide gels (SDS-PAGE) according to Laemmli [51]. After electrophoresis, gels were transferred to nitrocellulose paper (Hybond C + Extra, GE Healthcare, Buckinghamshire, UK). Membranes were stained with Ponceau S to confirm the transfer of proteins. In preliminary tests, two different saturation buffers were tested: 5% bovine serum albumin in TBS-Tween, Amersham ECL Prime Blocking Agent (GE Healthcare, Buckinghamshire, UK), and 5% nonfat milk powder in TBS-Tween. The last buffer allowed better results and was used in subsequent analysis.

The NOS-like protein detection was achieved with three different antibodies against mammalian nNOS from Santa Cruz Biotechnology, Inc: R20 (sc648), a rabbit polyclonal antibody raised against a peptide mapping near the C-terminus of rat nNOS; K20 (sc-1025), a rabbit polyclonal antibody raised against a peptide mapping at the N-terminus of human nNOS; H-299 (sc-8309), a rabbit polyclonal antibody raised against amino acids 2–300 mapping at the N-terminus of human nNOS. Anti-NOS antibodies were used at the final dilution of 1:250.

In order to normalize the WB results, the expression of housekeeping proteins was evaluated by a mouse monoclonal antibody raised against β-actin (diluted at 1:3,000; Santa Cruz Biotechnology, Dallas, TX, USA) and a rabbit polyclonal anti-glyceraldehyde-3-phosphate dehydrogenase (GAPDH) (diluted at 1:2500; ab37168, Abcam, Cambridge, MA, USA) antibodies. Anti-mouse IgG (A9044, Sigma-Aldrich, St. Louis, MO, USA) and anti-rabbit IgG (A9169, Sigma-Aldrich, St. Louis, MO, USA) peroxidase conjugate were used as secondary antibodies.

Rat brain homogenates were used as positive controls and negative controls were performed by the omission of primary antibodies. Detection was done using the Westar ηC Ultra (Cyanagen, Italy) and the band intensities were measured by Image J software (U.S. National Institutes of Health, Maryland). The normalization of WB results was obtained by the ratio between the quantified intensities of R20 and anti-GAPDH immunolabeled bands. In electrophoresis experiments, the “Amersham Full-Range Rainbow Molecular Weight Markers (12–225 KDa)” were used.

### 3.5. Immunofluorescence and Confocal Microscopy

Foot, tentacle, osphradium and nervous ring samples were collected and fixed in 4% paraformaldehyde (PFA) in phosphate-buffered saline (PBS, 0.01 M, pH 7.4) for 24 h, and cryoprotected in PBS containing 30% sucrose at 4 °C for at least 36 h. Samples were successively embedded in Killik solution (Bio Optica, Milan, Italy), frozen and cut into 20 μm transverse, sagittal or frontal sections in a cryostat, depending on the experiment. Consecutive serial sections were collected on microscope slides and sections were selected and processed for immunofluorescence. Briefly, sections were permeabilized with PBS plus 0.5% Triton-X-100 (PBS-T), blocked with 3% bovine serum albumin in PBS-T (PBS-BSA) for 30 min and successively incubated with the anti-NOS antibodies (R20 or H299 antibodies depending on the experiment) diluted 1:250 overnight at 4 °C. Sections were then incubated for 1 h at room temperature with the specific FITC- or CY3-conjugated secondary antibody depending on the experiment (diluted 1:50, Sigma-Aldrich, St. Louis, MO, USA). After several washes with PBS, sections were mounted on slides, cover-slipped, and observed with a fluorescence microscope. In controls, the primary antibody was omitted.

In double- or triple-labeling experiments, after incubation with the secondary antibody, sections were subjected to three washes for five minutes in PBS-BSA, incubated with phalloidin (rhodamine-labeled phalloidin, Molecular Probes, Eugene, OR, USA) diluted 1:200 in PBS-BSA for 2 h at room temperature and, after three further washes in PBS-BSA, incubated with the TOTO3 (TOTO^®^-3 Iodide, Molecular Probes^®^) diluted 1:500 in PBS-BSA for 30 min at ambient temperature. TOTO3 labels both DNA and RNA: the DNA staining allowed us to detect the position of the nucleus and the RNA staining together with the DIC images help in detecting the single cell shape and boundaries. The goat polyclonal anti Choline Acetyltransferase (AB144P, Millipore, Temecula, CA, USA) was used to characterized NOS-IR neurons of the pedal ganglia. Anti-rabbit FITC- (F6005, Sigma-Aldrich, St. Louis, MO, USA) or Cy3 (C2306, Sigma-Aldrich, St. Louis, MO, USA)-conjugated antibodies or anti-goat FITC-conjugated antibody (SC 2024, Santa Cruz Biotechnology, Dallas, TX, USA) were used as secondary antibodies depending on the experiment.

Epifluorescence images were captured on a Zeiss Axiophot microscope (Carl Zeiss Inc., Oberkochen, Germany) using a Photometric Sensys (Roper Scientific, Inc., München, Germany) digital camera. The low magnification IF images (shown in Figure 3A and Figure 5A) were obtained by a Axio Zoom V1.6 stereo zoom microscope (Carl Zeis Ltd., Oberkochen, Germany). Confocal microscopy observation was performed by a Leica TCS SP2 confocal microscope. The laser intensity and gain were set on the negative control that showed no signal. The same settings were used in sample observations.

### 3.6. RNA Extraction and RT-PCR Analysis

Total RNA was extracted using Trizol reagent (Invitrogen, Carlsbad, CA, USA) according to the manufacturer’s protocol. About one microgram total RNA of each sample was reverse-transcripted and amplified for the PCR using the Access RT-PCR System (Promega, Madison, WI, USA) with the following conditions: RT was carried out at 45 °C 45’ + 94 °C 2’ and PCR was done for 30/40 cycles at 94 °C 30”; 58 °C 30”; 68 °C 2’, with a final extension at 68 °C for seven minutes.

The oligos, previously successfully employed in *S. haemastoma* [20], were used: *sh*Nos_F (5′-129cgggacttctgtgacaccaacagatac-3′), *sh*Nos_R (5′-gaaggcgtgtttgaagatctcgcacag-3′), Actin_F (5′-catgaagtgcgacgttgaca-3′); Actin_R (5′-cacatctgctggaaggtgga-3′). Actin was used to normalize the intensity of the amplified fragment bands. Densitometric analyses of the bands were performed using software ImageJ 1.49r (Wayne Rasband, National Institutes of Health, Bethesda, MA, USA).

### 3.7. SOD and GPX Analysis

SOD activity was measured on 5 μL lysate with the SOD determination kit (Sigma #19160), according to the manufacturer’s instructions. GPX activity was determined on 20–50 μL lysate by an indirect assay based on the oxidation of glutathione (GSH) to oxidized glutathione (GSSG) catalyzed by GPX, which is then coupled to the recycling of GSSG back to GSH by glutathione reductase and NADPH (GPX cellular activity assay kit, Sigma #CGP1). Absorbances at 450 nm (for SOD activity) or 340 nm (for GPX activity) were recorded with a Tecan Infinite M200 microplate reader. SOD and GPX activity were normalized on the total protein content of samples, which was determined with the Bradford assay.

## 4. Conclusions

Immunohistochemical evidence reported in the present study strongly suggests that the constitutive *Sh*NOS is expressed by sensory and nervous cells. NOS-positive cells with characteristic features of sensory elements were detected in the osphradium, as well as in the tentacle and the foot. These cells were very similar to sensory cells retrogradely labeled by Lucifer yellow in the osphradium of *Aplysia californica* [52]. The generalized presence of NOS enzymes in putative sensory cells indicates a basic role for NO in the reception of external stimuli. Moreover, NOS expression at the synaptic level in all tissues sustains the role for NO in the modulation of synaptic functions in gastropods. Present findings also suggest that NO is somehow involved in pigment and mucous production and release. Given the intense NOS innervation of the foot epithelium, NO could also be involved in the modulation of foot gland secretion and cilia motility. In contract to vertebrates, NOS enzymes are not expressed in muscle cells of the mollusk.

Beyond the wide NOS distribution, the physiological relevance of the NOS/NO system in *S. haemastoma* is also indicated by thermal stress experiments showing that the *Sh*NOS gene, which is constitutively expressed, can be regulated by environmental factors acting as stressors for aquatic animals. These findings are in line with much evidence demonstrating that nNOS belongs to one of the main signaling pathways that mediates the neuronal response to different stresses [21].

## Abbreviations

am: acid mucin cell
BRN: basal retinal neurons
ca: central axis
CaM: calmodulin
CGC: cerebral giant cells
ChAT: choline acetyltransferase
CM: confocal microscopy
CNS: central nervous system
cr: ciliated region
cyt: cytoplasm
DTT: dithiothreithol
**eNOS** (NOS3): endothelial NOS isoform
ep: lamella epithelium
F: foot
GAPDH: glyceraldehyde-3-phosphate dehydrogenase
gc: ganglionic cell
glc: gland cells with a low affinity for hematoxylin
GPX: glutathione peroxidase
gr: glandular region
grv: lamella groove
IF: immunofluorescence
ils: interlamellar space
iNOS (NOS2): inducible NOS
l: lamellae
la: lamellar axis
m: muscle fibers
MGC: metacerebral giant cells
MW: molecular weight
NADPHd: NADPH diaphorase
nf: nerve fascicles
nNOS (NOS1): neuronal NOS isoform
NO: nitric oxide
NOS: NO synthase
NR: nerve ring
on: optic nerve
Os: osphradium
PBS: phosphate-buffered saline
PBS-BSA: 3% bovine serum albumin in PBS-T
PBS-T: PBS plus 0.5% Triton- X-100
PDZ: PSD-95/discs-large/ZO-1
PFA: paraformaldehyde
PR: pigmented region
R: rat
RL: rhabdomeric layer
S: Stutzzelle, supporting cell
Sch: Schleimzelle, mucous cell
*Sh*NOS: *Stramonita haemastoma* NOS isoform
Si1: Sinneszelltyp1, type 1 sensory cell
Si2: Sinneszelltyp 2, type 2 sensory cell
Si3: Sinneszelltyp 3, type 3 sensory cell
Si4: Sinneszelltyp 4, type 4 sensory cell
SOD: superoxide dismutase
sr: sensory region
Sz: Stutzzelle mit Zilien, supporting ciliated cell
T: temperature
Te: tentacle
WB: Western blot
